# A Physiologically Inspired Model for Solving the Cocktail Party Problem

**DOI:** 10.1007/s10162-019-00732-4

**Published:** 2019-08-07

**Authors:** Kenny F. Chou, Junzi Dong, H. Steven Colburn, Kamal Sen

**Affiliations:** Hearing Research Center, Department of Biomedical Engineering, Boston University, 44 Cummington Mall, Room 412, Boston, MA 02215 USA

**Keywords:** cocktail party problem, spatial tuning, sound segregation, cortical mechanisms

## Abstract

At a cocktail party, we can broadly monitor the entire acoustic scene to detect important cues (e.g., our names being called, or the fire alarm going off), or selectively listen to a target sound source (e.g., a conversation partner). It has recently been observed that individual neurons in the avian field L (analog to the mammalian auditory cortex) can display broad spatial tuning to single targets and selective tuning to a target embedded in spatially distributed sound mixtures. Here, we describe a model inspired by these experimental observations and apply it to process mixtures of human speech sentences. This processing is realized in the neural spiking domain. It converts binaural acoustic inputs into cortical spike trains using a multi-stage model composed of a cochlear filter-bank, a midbrain spatial-localization network, and a cortical network. The output spike trains of the cortical network are then converted back into an acoustic waveform, using a stimulus reconstruction technique. The intelligibility of the reconstructed output is quantified using an objective measure of speech intelligibility. We apply the algorithm to single and multi-talker speech to demonstrate that the physiologically inspired algorithm is able to achieve intelligible reconstruction of an “attended” target sentence embedded in two other non-attended masker sentences. The algorithm is also robust to masker level and displays performance trends comparable to humans. The ideas from this work may help improve the performance of hearing assistive devices (e.g., hearing aids and cochlear implants), speech-recognition technology, and computational algorithms for processing natural scenes cluttered with spatially distributed acoustic objects.

## Introduction

Our sensory systems are constantly challenged with detecting, selecting and recognizing target objects in complex natural scenes. In the auditory domain, the problem of understanding a speaker in the midst of others (a.k.a the “cocktail party problem,” or CPP) remains a focus of intensive research in a diverse range of research fields (Cherry 1953; Haykin and Chen 2005; McDermott 2009; Lyon 2010). Although hearing assistive devices and speech recognition technology have difficulties under CPP-like conditions, normal hearing listeners can solve the CPP with relative ease, indicating that a solution exists in the brain.

An impressive aspect of the CPP is the flexible spatial listening capabilities of normal-hearing listeners. A listener can broadly monitor (i.e., maintain awareness of) the entire auditory scene for important auditory cues, or select (i.e., attend to) a target speaker of a particular location. Indeed, the selection of the most relevant target often requires careful monitoring of the entire acoustic scene, and the ability to flexibly switch between these two states is essential in effectively solving the CPP. Yet, the neural mechanisms for this behavior remain unclear. Previous studies in birds and cats have suggested that spatially distributed sounds are segregated at the subcortical levels in the ascending auditory pathway (Konishi 2003; Yao et al. 2015). Though the specific differences in encoding across species remains an active research area, a key open question remains in both animals: how do flexible modes of listening emerge from spatially localized representations in the midbrain?

Behaviors of flexible tuning at the neuron level have been recently observed in the zebra finch field L (analog to the mammalian auditory cortex), and are dependent on their surrounding acoustic environment (Maddox et al. 2012; Dong et al. 2016). When target sounds (birdsongs) were presented separately from one of four spatial locations, cortical neurons showed broad spatial tuning, as indicated by similar discriminability performance across different target locations (Narayan et al. 2006). Remarkably, the neuron’s spatial tuning sharpened when a competing sound (song-shaped noise) was presented simultaneously from a different location. Such a neuron is sensitive to the spatial configuration of the target and the masker. A similar observation has been made in mammals, where spatial tuning is sharpened in the presence of multiple spatial streams (Middlebrooks and Bremen 2013).

Inspired by the recent discoveries in the cortical processing of spatially distributed sounds, we describe and implement a multi-stage model, which displays this flexible tuning characteristic, for processing multiple spatially distributed sound sources into a single audible acoustic output. The model is a generalization and application of a proposed model network for explaining the observations in the songbird field L (Dong et al. 2016). Our model assumes that spatial segregation is achieved at the midbrain level, and segregated sounds are either integrated or selected at the cortical level, corresponding to broad or sharp spatial tuning, respectively. The cortical model is realized in a spiking network. The processed spikes are then decoded via a linear stimulus reconstruction algorithm to produce audible waveforms. We demonstrate the flexible spatial tuning characteristic of the model, and compare its segregation performance with other engineering-based binaural sound segregation algorithms. The goal of this study is to build a physiology-based model inspired by the cortical responses in songbirds, and thereby demonstrate how the auditory system might operate under CPP-like conditions. The model provides a platform to explore physiologically relevant parameters for CPP processing, and provides an audible result that can be interpreted by human listeners, unlike the spike-distance-based discriminability measures presented in (Dong et al. 2016). The results generated using the model may motivate new physiological experiments to probe the auditory cortex, strategies for CPP processing in computer hearing, as well as strategies for processing cluttered visual scenes with computer vision.

## Methods

### Physiologically Inspired Algorithm

**Fig. 1:**
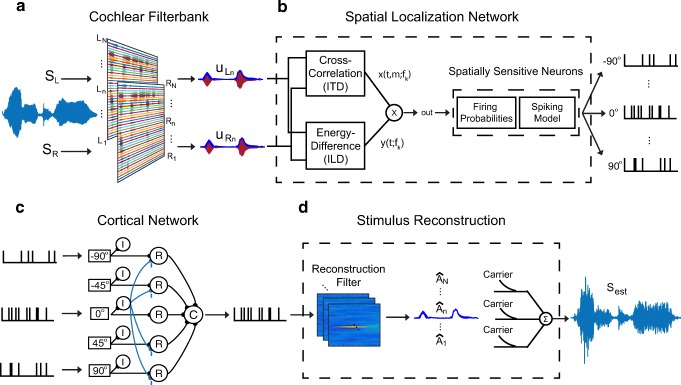
Detailed illustrations of the components of the physiologically inspired algorithm. **a** Cochlear filter bank: left and right channels of the input (*S*_*L*_ and *S*_*R*_) are filtered separately with an ERB gammatone filter bank. The outputs ${u}_{L_n}$ and ${u}_{R_n}$ illustrate the output of the *i*th frequency channel. The output signals’ envelopes are emphasized in blue. **b** Midbrain model: spatial localization network for one frequency channel. Left and right outputs from each frequency channel are used to calculate timing and level differences. A neuron spiking model then simulates a spiking pattern. Each frequency channel is processed by the network independently. **c** Cortical model: spikes from differently tuned spatial neurons act as the input to the cortical network, with an output showing the effects of lateral inhibition, which suppresses neural spikes from off-target spatial locations. Input layer: spatially tuned inputs at − 90, − 45, 0, + 45, and + 90. Middle layer: R, relay neurons and I, inhibitory interneurons. Output C, cortical neuron. The population of spikes from each frequency channel is processed by the cortical network independently. **d** Stimulus reconstruction. For each frequency channel, the reconstruction filter is used to calculate envelopes of sound ($\hat{A}\Big)$ from neural spikes from the cortical network. Carrier of the sound is added to the envelope by multiplication. The estimated sound from each frequency channel is summed to produce the final acoustic output (*S*_est_)

The Physiologically Inspired Algorithm (PA) is a sound processing algorithm that is based on the auditory system. It receives binaural speech inputs and transforms the speech signals into neural spikes for processing. After processing, it reconstructs the neural spikes back into the acoustic domain. The PA is composed of four key stages: a cochlear filter bank, a midbrain spatial localization model, a cortical network model, and a stimulus reconstruction step. These components are implemented in MATLAB (Mathworks, Natick MA) and the Python Programming Language, and are illustrated in Fig. 1. Below, we describe each of these components in details.

#### Cochlear Filter Bank

The cochlear filter bank represents a fundamental stage in the processing of sounds at the periphery of the auditory system, where sounds are first decomposed into different frequency bands (Patterson et al. 1992). This is implemented using an equivalent rectangular bandwidth (ERB) gammatone filter bank (Slaney 1998), a widely used representation in computational models of the auditory system (Fig. 1A). The filter bank consists of 36 frequency channels with center frequencies ranging from 300 to 5000 Hz. The PA uses 36 frequency channels because it provides a good balance between physiological accuracy and computational complexity. Additional frequency channels provided minimal benefit to the model. Subsequent stages of the model assume that each frequency channel is processed independently, thus the processing during each subsequent stages of the model are repeated for each frequency channel.

#### Midbrain Spatial Localization Network

To identify the location of the sound source, the auditory system exploits two important spatial cues: the interaural time difference (ITD) and the interaural level difference (ILD). ITD is created when a sound arrives at the more proximal ear earlier than the more distal ear, while ILD is created when the head shadows the more distal ear, decreasing the loudness compared with the more proximal ear. There are many models for binaural cue computation (Dietz et al. 2018). We elected to adapt a physiology-based model of the spatial localization network of the barn owl midbrain (Fischer et al. 2009), because it is one of the most accurate and best understood physiological systems for sound localization.

The model is illustrated in Fig. 1 B. It calculates ITD using a cross-correlation-like operation, and calculates ILD by taking the difference in the energy envelopes between the left and right signals. Readers are referred to the original work by Fischer and colleagues for detailed mathematical descriptions of binaural cue extraction (Fischer et al. 2009). In a subsequent stage of processing in the inferior colliculus model, the ITD cues are combined with ILD cues via a multiplication-like operation (Peña and Konishi 2001; Fischer et al. 2007). The model we adapted from Fischer et al. operates on discretized waveforms, while the next stage of our work (Fig. 1C) operates on neural spikes; therefore, we implement model neurons at this stage to encode the input waveforms into neural spikes. Five model neurons were implemented at this stage, with preferred ITDs and ILDs corresponding to − 90°, − 45°, 0°, 45°, and 90° azimuth, respectively. The firing probabilities for each of the model neurons are calculated by adding the ITD and ILD signals at the sub-threshold level followed by an input-output non-linearity given by a threshold sigmoid function. This overall operation effectively represents a multiplication of ITD and ILD cues as observed physiologically (Peña and Konishi 2001). Spikes are generated based on the calculated firing rates using a Poisson-spike generator. The calculations described above are done independently for each frequency channel, corresponding to the frequency channels of the cochlear filter bank model.

We tuned the specific parameters of the model neuron to match the ITDs and ILDs for a human head. We calculated the azimuth-specific ITD and azimuth- and frequency-specific ILD of KEMAR head-related transfer functions (HRTFs) for the five azimuth locations. For each preferred azimuth, we adjusted the ITD and ILD tuning parameters of the model neuron to match the ITD and ILD calculated for that azimuth and frequency.

#### Cortical Network Model: Inhibition Across Spatial Channels

The cortical network implements the critical computation of inhibiting off-target spatial channels. The network implemented here uses neural spikes as both input and output, and its architecture is illustrated in Fig. 1C. The five azimuth locations on the left side of Fig. 1C represent inputs from the midbrain model neurons. The inputs excite both relay neurons (R) and interneurons (I). The relay neurons for each azimuth excite a single cortical neuron (C) (i.e., cortical neuron integrates information across spatial channels), while the interneurons inhibit the relay neurons of other spatial channels. Each node of the network is composed of leaky integrate-and-fire neurons. For all neurons, resting potential was − 60 mV, spiking threshold was − 40 mV, and the reversal potential for excitatory currents was 0 mV. In relay neurons, the reversal potential for inhibitory currents was − 70 mV. In interneurons, synaptic conductance for excitatory synapses was modeled as an alpha function with a time constant of 1 ms. In relay neurons, synaptic conductance for both excitatory and inhibitory synapses were modeled as the difference of a rising and a falling exponential, where rise and fall time constants were 1 and 3 ms, and 4 and 1000 ms, respectively. Time constants were chosen based on the study by Dong et al. (2016), with the exception of the fall time of the relay neuron inhibitory synapses. Since the goal of this network is to optimize the reconstruction of the auditory target, the fall time of the relay neuron’s inhibitory synapse was increased to 1000 ms in order produce a strong, sustained suppression of maskers in off-target channels, to produce the best reconstruction of targets. An absolute refractory period of 3 ms was enforced in all neurons. Synaptic strengths were uniform across all spatial channels for the same type of synapse. The synaptic conductances between input to inter-neurons and relay neurons were 0.11 and 0.07 nS, respectively. The synaptic conductance from relay to cortical neurons was 0.07 nS. The conductance for the cross-spatial channel inhibition was 0.2 nS, which was the minimum value required to effectively suppress off-target spatial channels. The network connectivity was set to select sounds originating from 0° azimuth, as shown by the blue inhibitory pathways in Fig. 1C. Cortical network models are specific for each frequency channel, and the network structures are identical across all frequency channels. There are no interactions between frequency channels unless otherwise specified.

#### Stimulus Reconstruction

The output of the cortical network is a set of processed neural spikes for each frequency channel. In order to evaluate the model performance, the neural response was “translated” back to an acoustic waveform that humans can understand via a “stimulus reconstruction.” Here, we develop a novel stimulus reconstruction technique based on the estimation of a linear reconstruction filter (Fig. 1D) (Bialek et al. 1991; Stanley et al. 1999; Mesgarani et al. 2009; Mesgarani and Chang 2012). The basic idea is to first convolve a neural spike train with a reconstruction filter function to estimate the envelopes of the acoustic waveform (see “Optimal filter”). Since each frequency channel has a distinct set of neural spikes, this process is independently carried out for each channel. Then, the envelopes are used to modulate carrier signals to obtain narrowband signals. Finally, the narrowband signals across frequency channels are summed (without weighting) to obtain a reconstructed stimulus. We tested two different carrier signals for the reconstruction algorithm: (1) pure tones with frequencies equal to the center frequencies of each channel, and (2) band-limited noise limited to the frequency range for each frequency channel. In this manuscript, we present the results for pure tone carriers, which achieved the highest quantitative scores by the short time objective intelligibility (STOI, details in *Measures of Reconstruction Quality and Segregation Performance*, below) measure.

#### Optimal Filter

Commonly used analytically derived reconstruction filters assume that each frequency channel is independent of one another (Theunissen et al. 2001; Mesgarani et al. 2009): For a set of stimulus and response from frequency channel *f*, the stimulus waveform *s*_*f*_(*t*) can be reconstructed from a set of spike trains *x*_*f*_(*t*) with spike-times *t*_*i*_ (*i* = 1, 2, ···, *n*), by convolving *x*_*f*_(*t*) with a linear reconstruction filter, *h*_*f*_(*t*), to obtain an estimate of the original stimulus: ${s}_{est,f}(t)=\sum \limits_i^n{h}_f\left(t-{t}_i\right)$. We derive *h*_*f*_(*t*) in the frequency domain: $H\left(\omega \right)=\frac{S_{sx}\left(\omega \right)}{S_{xx}\left(\omega \right)}$, where *S*_*sx*_(*ω*) is the cross-spectral density of a training stimulus *s*(*t*) and the corresponding spike train *x*(*t*), and *S*_*xx*_(*ω*) is the power spectral density of the neural training response (Rieke et al. 1997; Gabbiani and Koch 1998). We restricted the length of *h*_*f*_(*t*) to 51.2 ms (or 2048 taps). The estimated original stimulus is then found by taking the unweighted sum across individual frequency channels: *s*_*est*_(*t*) = ∑_*f*_*s*_*est*, *f*_(*t*).

In contrast to the analytical approach described above, we introduced another frequency dimension, *ω*, to the optimal linear filter, to address the potential interactions across frequency channels: *h*_*f*_(*t*, *ω*). Such interactions may exist due to the relatively wide bandwidths of the gammatone filters, resulting in energies from one channel being picked up by adjacent channels. The estimated stimulus is obtained via a two-dimensional convolution obtained without zero-padding (“valid” mode in MATLAB or Python): *s*_*est*, *f*_ = *h*_*f*_(*t*, *ω*) ∗ *x*(*t*, *ω*), where *x*(*t*, *ω*) is the response spike trains for all frequency channels over time *t*. Since the convolution is only computed on elements that do not require zero-padding, the result is a one-dimensional signal of length (t–2048). To calculate *h*_*f*_(*t*, *ω*), we initialized a zero matrix and set *h*_*f*_(*t*, *ω*)|_*ω* = *f*_ = *h*_*f*_(*t*). We used gradient descent to minimize the mean-squared error (MSE) between the original signal’s envelopes and the reconstructed envelopes, treating the values of *h*_*f*_(*t*, *ω*) as free parameters. Initial one-dimensional reconstruction filters *h*_*f*_(*t*) were calculated in MATLAB, and two-dimensional filters were optimized using the Theano Toolbox in Python. The same process is repeated for each frequency channel *f*. We found that the optimal two-dimensional filter improved the reconstructions by 26 % relative to the one-dimensional filter, from 0.58 to 0.73, as assessed by the STOI measure (see “Measures of Segregation and Reconstruction Quality and Segregation Performance”).

#### Reconstruction Filter Training

We constructed a training waveform by extracting one instance of all call-sign and color-number combinations from the CRM corpus (see “Speech Stimuli”) and concatenated these into one continuous sound waveform. To derive the optimal filter, the training waveform was presented to the PA at 0° as the training input stimulus, and the corresponding cortical response was used as the training target. Since the optimal filter is a mapping between the clean CRM utterances (prior to introducing HRTF) and neural spikes, the effect of HRTF are removed from the reconstructed stimuli. After deriving the reconstruction filter, we tested the algorithm on other CRM sentences and their corresponding neural responses. Note that the training phase only requires training on clean speech. The filter was not re-trained as long as frequency channels remain independent of one another.

#### Cross Validation and Overfitting

We ran simulations with randomly selected TIMIT corpus sentences (Victor et al. 1990) while reconstructing with the CRM corpus-trained reconstruction filter. The reconstruction performance (see “Measures of Reconstruction Quality and Segregation Performance”) did not differ significantly from the simulations ran with the CRM corpus, differing by 4 % on average. Based on this result, we determined that overfitting was not an issue.

#### Code Accessibility

The code for the PA is available upon request.

### Simulations

#### Speech Stimuli

The coordinated response measure (CRM) corpus (Bolia et al. 2000) was used to train and test the novel stimulus reconstruction technique, as well as test the segregation and reconstruction results using our physiology-inspired model. The CRM Corpus is a large set of recorded sentences in the form of [Ready CALLSIGN go to COLOR NUMBER now], where call sign, color, and number have 8, 4, and 8 variations, respectively. All recordings were stored as 40 kHz binary sound files. Directionality was added to the recordings by convolving each recording with KEMAR (Burkhard and Sachs 1975) head-related transfer functions (HRTFs) corresponding to the appropriate location (Gardner and Martin 1995; Kim and Choi 2005). For each simulation, we randomly selected three sentences from the CRM corpus, and designated one to be the “target” and the remaining to be the “maskers.” Since the result of the simulations is quantified using entire sentences, having repeats in any of the three keywords may artificially inflate the results. Therefore, sentences in each trio cannot contain the same call sign, color, or number. For simulations with single talkers, only the “target” sentence is used.

#### Simulation Scenarios

To test the segregation and reconstruction quality of the PA, we configured the model network to “attend to” 0° azimuth (Fig. 1C) by only activating the inhibitory neuron in the 0° spatial channel. This is achieved by setting the I-R connection matrix such that only the non-zero values in the matrix correspond to the connections from 0° I neurons to other spatial channels’ R neurons.

We designed three simulations to demonstrate that the PA is capable of: (1) monitoring the entire azimuth in quiet, (2) selectively encoding a preferred location while suppressing another non-preferred location when competition arises, and (3) robustly encoding a preferred location when maskers became louder than targets. Each simulation was repeated 20 times, each time using a different set of CRM sentence trios. In the first simulation, we presented the PA with a single target at locations between 0 to 90° in azimuth, at 5° intervals. We then calculated assessment measures (see “Measures of Reconstruction Quality and Segregation Performance”) of the quality and intelligibility of the reconstructed signal compared with the original target signal. In the second simulation, we presented one sentence at the target location (0°) and two masker sentences at symmetrical locations in 5° intervals from 0 to ±90°. The sentences have a target-to-masker ratio (TMR) of 0 dB, defined as the energy difference between the target and individual maskers. We then calculated speech intelligibility of the reconstruction compared with the target and masker sentences, respectively, for all masker locations. We then swapped the location of the two masker sentences and repeated the simulation. The third simulation was designed to test the robustness of the PA at low SNRs. In this simulation, the target was fixed at 0° and the maskers fixed at ± 90° respectively. The TMR was then varied between −13 and 13 dB. This equates to signal-to-noise ratios (SNRs) of −16 to 10 dB.

#### Measures of Reconstruction Quality and Segregation Performance

We compared several objective measures of speech intelligibility and quality including the STOI (Taal et al. 2010), the normalized covariance metric (NCM) (Chen and Loizou 2011), and the PESQ (Rix et al. 2001), each of which calculates the intelligibility and quality of a processed signal compared with its original unprocessed form (i.e., a reference signal). A higher score indicates better intelligibility or quality of the processed signal to human listeners. All CRM sentences have the words “ready,” “go to,” and “now,” and there is a possibility that these repetitions would inflate individual objective scores. Therefore, a relative segregation performance is quantified by the score difference between using target versus maskers as the reference signal, which we call Δ. In our analyses, all three objective measures performed similarly, in a qualitative sense. We present only the STOI results here. The STOI is designed to measure the intelligibility of speech in the presence of added noise, which makes it an appropriate measure to quantify the quality of the reconstructed speech. STOI scores are shown to be well-correlated to subjective intelligibility (Taal et al. 2010):

$$ \mathrm{Predicted}\ \mathrm{subjective}\ \mathrm{intelligibilit}y\ \left(\%\right)=\frac{100}{1+\exp \left(-13.1903\bullet STOI+6.5192\right)} $$

where subjective intelligibility score is measured by the percent of words correctly recognized by human listening subjects. By this measure, an STOI score above 0.7 is highly intelligible, corresponding to 90 % correct. MATLAB functions for computing intelligibility measures were generously provided by Stefano Cosentino at the University of Maryland.

#### Frequency Tuning

The model network we used assumes sharp frequency tuning, where frequency channels do not interact with one another. Cortical neurons have been found to have various sharpness in frequency tuning (Sen et al. 2001), and the model performance may depend on frequency tuning width. For these reasons, we explored the effects of frequency tuning curves on the network performance for single-target reconstructions. We modeled the spread of information across frequency channels with a Gaussian-shaped weighting function, centered around the center frequency (CF) of each frequency channel:

$$ {w}_{i,j}=\exp \left(-\frac{{\left(C{F}_j-C{F}_i\right)}^2}{2{\sigma}_i^2}\right) $$

where *i* and *j* are the indices of frequency channels, and *σ* is the standard deviation. The spread of information is modeled by having the relay neurons centered at *CF*_*i*_ receive inputs from its neighboring frequency channels, centered at *CF*_*j*_,weighted by *w*_*i*, *j*_. The values of *σ*_*i*_ used in this simulation was determined by introducing the variable *Q*, defined as the ratio of CF to the full-width at half-maximum (FWHM) of a tuning curve (Sen et al. 2001). Here, we formulate *Q* in terms of the Gaussian weighing function’s FWHM, which can then be related to *σ*_*i*_: $Q=\frac{C{F}_i}{FWHM}=\frac{C{F}_i}{2\sqrt{.2\ln (2)}{\sigma}_i}$. We tested *Q*s ranging from *Q* = 0.85 (broad tuning) to *Q* = 23 (sharp tuning). For reference, *Q* values from field L in the zebra finch have been reported to range from 0.4 and 7.8 (Sen et al. 2001). This is the only simulation where there are interactions between frequency channels. Due to this cross-frequency interaction, we re-trained the reconstruction filter for each *Q*, using the same training sentences previously described as the training stimulus, the corresponding spike trains for each *Q* as the training target.

#### Robustness to Frequency Tuning

We processed 20 target sentences, placed at 0° azimuth, with our model network for *Q* ranging from 0.85 to 23. Performance of the model at each *Q* was evaluated by the intelligibility of the reconstructions with the targets alone, quantified by the STOI score (Table 1).

**Table 1:** Effect of *Q* on single-target reconstructions

*Q*	STOI	±
0.85	0.3567	0.0536
1.3	0.4902	0.0425
2.5	0.5930	0.0336
3.4	0.6459	0.0255
5.1	0.6671	0.0270
7.6	0.6805	0.0241
10.6	0.6723	0.0254
23.4	0.6906	0.0243

#### Engineering Algorithms

Although our main goal here was to develop a physiologically inspired model, we were curious to compare the segregation performance of the PA to cutting-edge engineering algorithms. Some engineering algorithms, notably beam-formers, rely on increasing the number of sound inputs with the number of sources (see comparative discussion in Mandel et al. 2010), and/or rely on monaural features, such as pitch (Krishnan et al. 2014). In contrast, the PA is a binaural algorithm requiring only two inputs (left and right ear signals), as in the human auditory system, and does not use any additional information from monaural features. Thus, for a controlled comparison, we compared the segregation performance of the PA with two cutting-edge engineering algorithms that were essentially binaural: model-based expectation-maximization source separation and localization (MESSL) and a deep neural network (DNN) trained with binaural cues, and evaluated all algorithms with the same STOI metric.

#### MESSL

MESSL algorithm by the Ellis group is freely available via Github (https://github.com/mim/messl) (Mandel et al. 2010). MESSL uses binaural cues to localize and separate multiple sound sources. Specifically, it uses a Gaussian mixture model to compute probabilistic spectrogram masks for sound segregation. For the best performance possible, the correct number of sound sources in each scenario was provided to the algorithm as a parameter. MESSL also requires a parameter *tau*, which is an array of possible source ITDs converted to numbers of samples. For this parameter, we used 0 to 800 μs of ITDs, and omitted the negative taps. MESSL does not require any training, but does require careful selection of these parameters to optimize performance. We systematically searched over a range of *tau* and selected the ones that yielded the highest STOI and PESQ scores for this study.

#### DNN

The DNN algorithm is also freely available online (http://web.cse.ohio-state.edu/pnl/DNN_toolbox/) (Wang et al. 2014). DNN isolates a target sound from noisy backgrounds by constructing a mapping between a set of sound “features” and an ideal spectrogram mask. For a controlled comparison with the other algorithms, we replaced the monaural features in the DNN algorithm with three binaural features: ITD, ILD, and interaural cross-correlation coefficient (IACC). ITD was calculated through finding the peak location of the time-domain cross-correlation function, and the IACC was the peak value. To be consistent with the features used by the DNN model reported by Jiang et al. 2014, 64 frequency channels were used and the features were calculated for each time-frequency unit. We trained the DNN with sentences from the CRM corpus. Often, a critical factor in optimizing the performance of the DNN is the amount of training data used. The number of training sentences needed for the DNN performance to reach the highest performance under the two-masker scenario described above was used to train the DNN.

#### Stimuli for Comparing Algorithms

Sentences from the CRM corpus described above were used to form the speech stimuli for all three algorithms. The input sound mixtures had target-to-masker-ratios (TMRs) of 0 dB. TMR is defined as the sound level of the target to a single-masker, regardless of the number of maskers present.

#### Scenarios for Comparing Algorithms

Two scenarios were simulated for all three algorithms: In the first scenario, a target stimulus was placed at 0° while two symmetrical maskers were varied between 0 and ± 90°. In the second scenario, a target stimulus was placed at 0° while 2, 3, or 4 maskers were placed at ± 45 and/or ± 90° in all possible combinations. The STOI score was then calculated for each condition. For MESSL and DNN, STOI was calculated by comparing the output of each simulation against each of the original individual stimuli.

#### Segregation Comparison

Here, we describe the measure of segregation performance in more details. As mentioned previously, we quantify the segregation performance as the difference between STOIs, computed using either target or masker as the reference waveform, or ΔSTOI. The output of the PA is noisy due to the stimulus reconstruction step, resulting in an artifact that is absent in MESSL and DNN. The use of ΔSTOI allows comparison of true segregation ability, independent of reconstruction artifacts.

#### Algorithm Training

MESSL did not require any training. The amount of training versus performance for the DNN algorithm was determined experimentally using scenario 2. DNN required training on targets in the presence of maskers, with about 100 sentence mixtures required to reach peak performance, which was used to train the DNN algorithm in the simulations. Due to the small number of sentence available in the CRM corpus, we recognize we have most likely overfitted the DNN. However, we do not believe this to be an issue, because we want to create the best possible performance for DNN. The PA was trained in the same manner as previously described in “Reconstruction Filter Training.”

## Results

### A Physiologically Inspired Algorithm for Solving the CPP

**Fig. 2:**
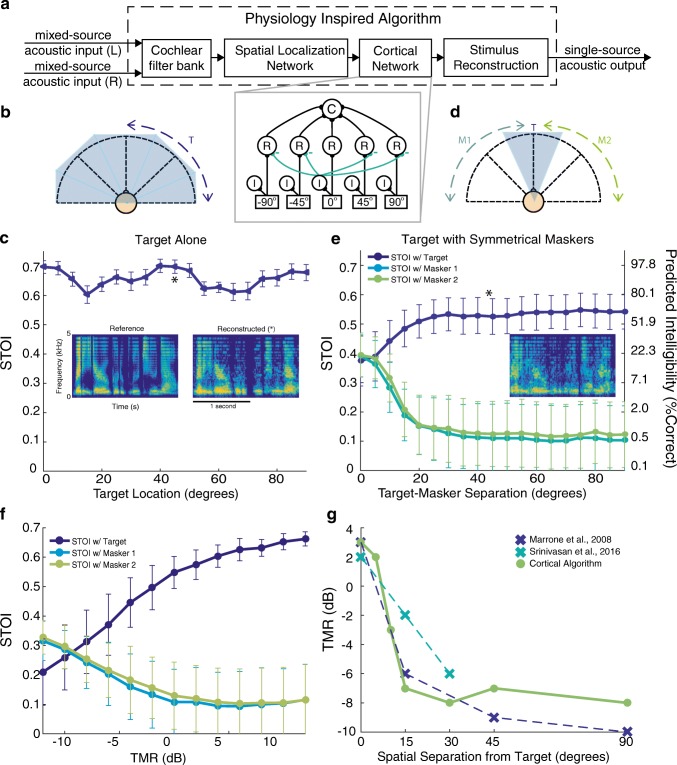
The physiologically inspired algorithm. **a** Flow diagram of the algorithm. Dual channel (L and R) audio is used as the input to CA. The CA consists of a cochlear filter bank, a spatial localization network, the cortical network and a stimulus reconstruction algorithm (see “Methods”). The output is a single acoustic waveform. **b**–**e** The performance of the CA with a frontal beam-former cortical network where 0° inhibits all other spatial channels (inset). **b** Monitor mode simulation with the target alone roved from 0 to 90°. **c** Simulation results of **b**: short-time objective intelligibility measure (STOI) as a function of location is shown. Insets show the spectrograms for the vocoded target and the reconstructed output for the target position of 45° indicated by the asterisk. **d** Selective mode: the target sentence is fixed in the front, while two maskers are placed symmetrically at angles from 0 to ± 90°. **e** Simulation results of **d** the STOI of the reconstructed output is computed with both the target and masker signals as reference. Inset shows the spectrogram of the reconstructed output for when the maskers are positioned at ± 45° (indicated by the asterisk). The y-axis on the right-hand side shows the predicted intelligibility that corresponds to the calculated STOI scores. **f** Robustness of the CA. STOI curves with respect to target and maskers vs. target to masker ratio (TMR). **g** Comparison with psychophysics

We built upon the network model for cortical responses, as described above, to design a physiologically inspired algorithm (PA) to process human speech in a CPP-like setting (Fig. 2A). The input to the PA was binaural speech input, corresponding to the sound signals at the left and right ears. The PA was composed of a cochlear filter bank, a midbrain spatial localization network, a cortical network, and a stimulus-reconstruction algorithm. The biological motivation and the computations carried out in each of these processing stages are described in details in the “Methods” section, and a detailed figure for each stage of the model is illustrated in Fig. 1.

### Frequency Tuning

Cortical neurons can be tuned to a variable range of frequencies. This tuning property differs across both species and cell types. We tested the effect of frequency tuning of model cortical neurons on the intelligibility of the model output for single CRM sentences, and found that sharper frequency tuning (higher *Q* values) correspond to higher intelligibility (Table 1). In our model, when *Q* is greater than 23.4, frequency channels essentially become independent of one another.

### Performance: Monitoring Mode

The performance of PA is similar to the responses of cortical neurons in the avian auditory cortex. The monitor mode of the network is illustrated in Fig. 2B. When a target alone was roved in space, the reconstructed signal resembled that target regardless of location, as demonstrated by similar STOI scores across all locations (Fig. 2C). The similarity between the target and reconstructed waveforms is also visually illustrated in their spectrograms (Fig. 2C, inset). Such encoding of single sources at all locations would allow the listener to broadly monitor the entire acoustic scene.

### Performance: Selective Mode

The selective mode of the network, in the presence of competing sounds, is illustrated in Fig. 2D. For these simulations, the target was fixed at 0°, while two maskers of equal amplitude (0 TMR) were played simultaneously and symmetrically at angles anywhere between 0 and ± 90°. The PA was used to segregate and reconstruct the frontal target while suppressing the maskers. Three STOI curves are shown (Fig. 2E): The dark blue curve represents the STOI scores calculated using the target as the reference waveform. Error bars indicate standard deviation. The STOI score increases as a function of spatial separation, and plateaus at around 25°. The teal and green curves represent the STOI scores calculated using either masker 1 or masker 2 as the reference waveform, respectively. These curves show the opposite trend as the target-referenced STOI. The STOI measure demonstrates that the PA is able to segregate the target signal effectively when the maskers are separated by more than about 15° from the target, illustrated by the separation between the dark blue and the teal and green curves. The configuration of the network was not changed between the quiet (target alone) and noisy (target + masker) conditions, indicating that the same network achieved both broad tuning to single targets and sharpened tuning in the presence of maskers.

### Robustness

We found that the PA remains effective in challenging situations where the intensity of the target source is weaker than the maskers. To demonstrate this robustness, we presented the target at 0°, and two maskers at ± 90°, and varied the target to masker ratio (TMR). We found that the reconstructed signal more closely resembles the target than maskers down to about − 5 dB, as reflected by higher STOI when comparing the reconstruction to the target vs. the maskers (Fig. 2F).

### Comparison with Human Psychophysics

In psychoacoustics, the benefit of increased separation between target and masker has been termed spatial release from masking (SRM). Previous studies have recorded the TMR thresholds for 50 % correct human performance in listening experiments with a center (0°) target and symmetrical maskers at different spatial separations (Marrone et al. 2008; Srinivasan et al. 2016). For comparison, we calculated the 50 % classification threshold based on STOI for each target-masker separation. The 50 % classification threshold for each separation was the TMR where the intelligibility measures (STOI) of the algorithm was higher compared with the target sentence than the masker sentences for at least 50 % of sentences. Figure 2G compares the 50 % TMR thresholds of PA with those measured for humans in psychoacoustic studies. The overall range and trend of performance of the PA was qualitatively similar to human performance.

### Different Configurations of Cortical Network

**Fig. 3:**
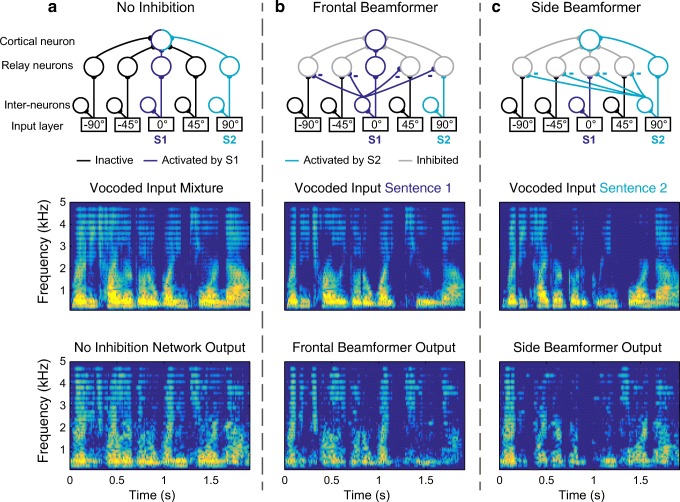
Different configurations of the cortical network. For all simulations, two sentences were presented from 0° (S1) to 90° (S2) simultaneously. **a** With no cross-spatial channel inhibition, the cortical network is broadly tuned and the reconstructed stimulus resembles the mixture of both sentences (spectrograms in middle and lower panels). **b** With a frontal beam-former cortical network, the reconstructed stimulus resembles the sentence presented from front (S1). **c** With a side beam-former network, the reconstructed stimulus resembles the sentence presented from 90° (S2)

In the previous section, we simulated a frontal “beam-former” cortical network where the 0° azimuth frontal channel inhibits all other channels. The cortical network model can be configured to different spatial preferences by changing the cross-spatial-channel inhibitory connections. Figure 3 demonstrates how changing the inhibitory connectivity of the cortical network while using the same mixed-source inputs changed the reconstructed signal. For these simulations, two sentences (S1 and S2) were presented simultaneously from the front (S1, 0° az) and right side (S2, 90° az). With cross-spatial-channel inhibition turned off, the reconstructed waveform resembled the mixture of the two sentences (Fig. 3A). With a frontal beam-former network, the reconstructed waveform resembled the sentence in the front (Fig. 3B). In this configuration, *STOI*_output, *S*1_ = 0.57, while *STOI*_output, *S*2_ = 0.10. With a different configuration of inhibitory connections as shown in the side beam-former network, the reconstructed waveform resembled the sentence on the side (90°) (Fig. 3C). In this configuration, *STOI*_output, *S*1_ = 0.09, and *STOI*_output, *S*2_ = 0.63. Thus, depending on the configuration of the inhibitory connections, the network outputs resemble sound streams originating from specific spatial locations.

### Comparison with Cutting-Edge Engineering Algorithms

There have been intensive efforts to perform auditory source segregation using purely engineering algorithms (Wang and Brown 2006). Although our main goal here was to develop a physiologically based model, we were curious to compare the segregation performance of the PA with two cutting-edge engineering algorithms: model-based expectation maximization source separation and localization (Mandel et al. 2010) and a deep neural network (Jiang et al. 2014; Wang et al. 2014) trained with binaural cues; both were evaluated with STOI.

**Fig. 4:**
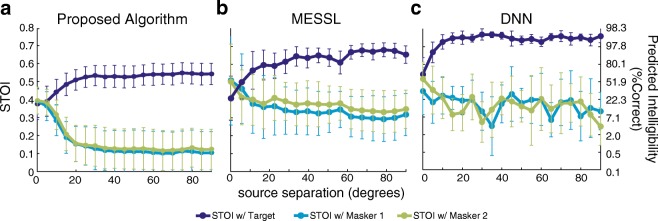
Comparison of segregation performance. The two symmetrical-masker simulation was carried out in the **a** PA, **b** MESSL, and **c** DNN algorithms. The STOI of each algorithm output was quantified using the target sentence (dark blue line) or masker sentences (green and light blue lines) as references. STOI scores are shown on the left hand y-axis, and the corresponding predicted intelligibility (as percent correct), calculated using the conversion from (Taal et al. 2010), are shown on the right hand y-axis.

Figure 4b and c show the STOI curves for MESSL and DNN. Ideally, for good segregation, STOI values relative to the target should be as high as possible, while STOI values relative to the masker should be as low as possible. Target STOI were higher for the DNN and MESSL, compared with the PA (Fig. 4). However, the Masker STOI values were also higher for the DNN and MESSL, compared with the PA. Since STOI is designed to measure relative intelligibility, it alone is not a good measure of segregation. To quantify the segregation of the target and masker, we computed the difference in the STOI values, ΔSTOI, as well as the differences in their corresponding intelligibility values (see “Methods”). The PA had higher ΔSTOI values compared with MESSL and DNN, and lower ΔIntelligibility (Table 2).

**Table 2:** Differences in objective scores between the proposed algorithm and two other engineering algorithms for sound source segregation

	ΔSTOI	ΔIntelligibility
Proposed algorithm	**0.394**	69.7
MESSL	0.276	76.8
DNN	0.325	**84.3**

## Discussion

In this study, we have developed a novel, physiologically inspired algorithm (PA) for processing sounds in a CPP-like setting. Our results demonstrate that, like cortical neurons in birds and mammals, the PA is capable of operating in two distinct modes. In the presence of single sounds, the algorithm reconstructs sounds across the entire azimuth, effectively monitoring the acoustic space (Fig. 2D). Such a behavior may also be important in complementing the visual system in detecting objects, when an object is outside of or in the periphery of the visual field. In the presence of multiple competing sounds, with cross-spatial channel inhibition in the cortical network, the algorithm segregates a target sound from a mixture, allowing the network to selectively listen to a target (Fig. 2E). Note that with cross-spatial channel inhibitory connections in place, switching between the two modes only requires the presence or absence of a competing sound, not a reconfiguration of the cortical network. The PA is robust to the level of the masker relative to the target (Fig. 2F), and displays trends similar to human performance (Fig. 2G). The flexible behavior of the PA reflects every-day behavior of humans and animals, but this important feature is non-existent in current sound-processing technologies. Thus, the ideas from the PA, the first physiologically-based algorithm to exploit binaural spatial cues and utilize passive, flexible tuning behavior to solve the CPP, may help improve the performance of a broad range of sound processing systems and devices that have difficulty under CPP-like conditions.

### Physiological Basis and Implications

Our model is a synthesis of multiple stages of auditory processing that are based in physiological mechanisms of birds. The spatial localization network was based the barn-owl’s midbrain, one of the best understood model systems for spatial localization. The cortical level was based on experimental recordings from field L, the analogue of primary auditory cortex, in the zebra finch. Although the cortical level in birds is structurally different from the mammalian cortex, recent studies have shown that the functional properties of auditory neurons in field L are remarkably similar to mammalian cortex (Calabrese and Woolley 2015). The spatial responses of field L neurons (Maddox et al. 2012) are also similar to neural responses in the primary auditory cortex of cats, which showed broad spatial tuning for single sound sources but sharper spatial tuning in the presence of two competing auditory streams from different locations (Middlebrooks and Bremen 2013). Therefore, it is possible that the mechanisms in the avian-based PA model also exist in mammals. We are currently conducting experiments in mice to determine whether the A1 neurons also display flexible tuning behavior.

### Effects of Spatial Tuning Width and Frequency Tuning Width

The model as implemented here relies on tuned responses to both spatial location and frequency. A previous study has investigated the effect of sharpness of spatial tuning in a similar network model (Dong et al. 2016) and found that the performance of the network remained robust over a broad range of spatial tuning widths. Specifically, for tuning curves modeled as Gaussians, performance was robust for tuning curve widths (i.e., twice the standard deviation) ranging from less than 15° up to 80°.

In mammalian systems, spatial tuning curves for single locations have been found to be broadly tuned “open-ended” response functions, e.g., a sigmoidal function of location, instead of the spatially tuned channels employed in our model, experimentally observed in avian species. Computational modeling shows that it is possible to construct accurate readouts for spatial location either using sharply tuned circumscribed receptive field (e.g., Gaussian) or broadly tuned “open-ended” response pattern (e.g., a sigmoid) (Lee and Groh 2014). Thus, the readout computation for spatial location for single sound sources may be different in birds and mammals. However, once the readout for single locations has been achieved, cross-spatial-channel inhibition could be used as described in our model, to achieve hotspots on the spatial grid. Our preliminary data, using a very similar experimental paradigm have revealed similar spatial grids in the mouse (Gritton et al. 2017). This suggests that despite potential differences in readouts for single sounds, avian and mammalian species may show similar cortical representations for sound mixtures.

To evaluate the effect of frequency tuning widths on reconstructed outputs, we simulated cortical networks with gradually increasing cross-frequency connectivity profiles (see “Methods”), which would broaden the effective frequency tuning widths of individual channels, and evaluated the performance of our model for capturing a single target sentence placed at 0° azimuth. We found that performance of our model network remained robust over a range of connectivity widths (*Q* factors 23.5–3.5) and degraded for lower values (Table 1). The range of *Q* values over which performance remained robust contains values similar to those observed in the avian auditory cortex (Sen et al. 2001), suggesting that physiologically observed values for frequency tuning width can support robust stimulus reconstruction.

### Bottom-up vs. Top Down Processing

In our model, some of the critical components of the overall computation, e.g., peripheral filtering of sounds and spatial localization using acoustic cues such as ITD and ILD, occur prior to the cortical level, supporting the idea that bottom-up processing plays an important role in separating auditory mixtures into different spatial streams. The cortical level then builds on these computations to select appropriate stream(s) to attend. The brain combines both bottom-up and top-down processes to solve the CPP (Bee and Micheyl 2009). Modeling top-down processes would be an appropriate next step in extending our model.

### Relating to the Visual System

Cross-spatial-channel inhibition plays an important role in the cortical network of our proposed model. In our model, inhibitory connections are recruited when multiple competing objects are simultaneously present at different locations. This is reminiscent of an interesting finding in the primary visual cortex, where simultaneously stimulating the classical and the non-classical receptive field of a visual cortical neuron increases the sparseness, reliability, and precision of neuronal responses, by recruiting strong “cross-channel” inhibition (Vinje 2000; Vinje and Gallant 2002; Haider et al. 2010). Thus, similar inhibitory cortical circuitry may be involved in processing complex scenes in both the auditory and the visual cortex. Unlike primary auditory cortical neurons, primary visual cortical neurons have much more spatially localized receptive fields. However, spatial receptive fields in downstream visual areas thought to be involved in object recognition, e.g., inferotemporal cortex (IT), are physically much larger. Interestingly, when a second stimulus is presented in the visual receptive field of IT neurons, neurons can be suppressed by or tolerant to the second stimulus (Zoccolan et al. 2007). The observations from these studies suggest that the principle of the PA could also be applied to the visual cortex. In a visual scene cluttered with multiple objects, the cortical model would allow inhibitory neurons with spatial receptive fields containing a target object to suppress the responses of excitatory neurons with receptive fields in other spatial locations.

### Comparison with Engineering Algorithms

There have been intensive efforts to solve the CPP using computational models for auditory scene analysis (Wang and Brown 2006). To our knowledge, there are no other physiological models for auditory scene analysis that exploit the remarkable spatial processing capabilities of the auditory system. We compared the PA and two other algorithms that also use binaural cues to perform sound segregation—MESSL and DNN.

Although all three algorithms were binaural, some differences are worth noting. The performance of DNN was heavily dependent on the amount of training sentences available. To achieve high performance using the small number of CRM sentences available, it was necessary for us to severely over-train the algorithm. In addition, it is noteworthy that the training set for DNN were target-in-maskers, meaning that the testing set was included in the training set. On the other hand, the training set for PA consists of one single modified target sentence. It is unknown how well the DNN generalizes to different target and masker locations after training, whereas the PA can be configured to varying spatial configurations of target and maskers by adjusting the inhibitory connectivity in the cortical network, without further training (Fig. 2). One advantage of the MESSL algorithm is that it did not require any training; however, it was the most sensitive of all the algorithms to increasing number of maskers. It also required an initial estimation of the number of sources present to accurately separate a sound mixture, making it difficult to implement in real-world settings. Another important distinction between the algorithms is that the PA is rooted in physiology, whereas MESSL and DNN are not. The cortical network architecture enables the PA to be used as a predictive tool to motivate new experiments in neuroscience, and novel experimental findings can be incorporated into the PA.

The comparison shown in the results is intended as an additional step in evaluating the PA’s performance, not as a full benchmarking, which is outside the scope of this study. Aside from constraining all the algorithms to be “binaural-only,” we did not change the model structure or the parameters for MESSL and DNN (see “Methods”). Thus, it is possible that the relative performance of these algorithms could be improved further by adjusting their parameter values, or operating on different kind of sound mixtures. Depending on the context and objective measure used, our results demonstrate that the segregation performance of the PA can be either better or worse than the state-of-the-art engineering algorithms (Table 2). In the next section, we discuss some limitations to the PA and how its performance could be improved.

### Limitations

Aside from the training, two notable factors greatly impact the performance of the PA. The PA relies on the midbrain model to perform spatial segregation of sound mixtures. Therefore, its segregation performance depends on the correct computation of binaural cues and the localization accuracy of the midbrain model. The correct calculation of binaural cues is, in turn, dependent on the number of sources present and the signal-to-noise ratio. This bottleneck is unavoidable without using additional cues (e.g., monaural cues to aid in the grouping of sounds). The second big factor is stimulus reconstruction. The process of stimulus reconstruction results in noisy artifacts, and severely degrades the reconstruction sound quality and intelligibility. This may explain why even though ΔSTOI of PA is higher than the two algorithms compared here, its intelligibility scores are much lower (Table 2). We suspect that the performance of the PA as a sound segregation algorithm can be improved by addressing this point.

### Conclusion and Future Directions

Many other facets of complex cortical circuitry and processing exist, e.g., laminar processing (Atencio and Schreiner 2010a, 2010b). These processes have also been shown to play important roles in solving the CPP. Monaural processing, such as temporal coherence (Elhilali et al. 2009; Shamma et al. 2011) and pitch tracking, has been frequently used in engineering algorithms for CPP processing. Experiments have shown that attention (i.e., top-down control) also plays an important role (McDermott 2009; Bronkhorst 2015). Although the exact neural mechanisms responsible for each of these processes are still not entirely clear, one can imagine modeling each process in a spiking neural network, each of which can be linked to the spatial processing model described here to construct a comprehensive physiological model of CPP processing. For now, we have not included any of these other processes because it is beyond the scope of this work.

Our focus in this study was on the spatial response properties of the cortical neurons in CPP-like settings. In this work, we demonstrated that the PA can adjust its spatial tuning based on whether competing objects are present in the auditory scene. We tested its performance as a sound segregation algorithm, compared these performances to other, non-physiology-based algorithms, and suggest how it can be improved. We also discussed its biological implications and how its principles can be used to model other experimental observations (i.e., in the visual cortex).

The interneurons in our cortical model play a critical role in realizing the flexible spatial tuning behavior of the PA. Does the same network architecture exist in mammals? If the interneurons were silenced, e.g., with optogenetics, will the animal be unable to solve the CPP? We plan to extend our experimental paradigm to the mouse model, where the availability of optogenic tools enables us to conduct appropriate experiments to answer these questions.
